# Novel Inhibitor Discovery of *Staphylococcus aureus* Sortase B and the Mechanism Confirmation via Molecular Modeling

**DOI:** 10.3390/molecules23040977

**Published:** 2018-04-23

**Authors:** Guizhen Wang, Xiyan Wang, Lin Sun, Yawen Gao, Xiaodi Niu, Hongsu Wang

**Affiliations:** College of Food Science and Engineering, Jilin University, Changchun 130062, China; wangguizhen510@126.com (G.W.); wangxiyan199294@163.com (X.W.); sunlin16@mails.jlu.edu.cn (L.S.); gaoyw16@mails.jlu.edu.cn (Y.G.); niuxd@jlu.edu.cn (X.N.)

**Keywords:** *Staphylococcus aureus*, coptisine, sortaseB, cytotoxicity, adhesion, molecular simulation

## Abstract

SortaseB (SrtB) plays a critical role in *Staphylococcus aureus* (*S. aureus*) infections. According to the reports in the literature, SrtB can anchor the IsdC to the cell wall to capture iron from the host to achieve a successful invasion. On the other hand, SrtB could also affect the adhesion of *S. aureus* to host cells based on previous studies. Here, we report about a novel SrtB inhibitor, coptisine, a natural compound that does not exhibit antibacterial activity but can inhibit the SrtB activity in vitro. A cytotoxicity test indicated that coptisine protects human lung epithelial cells from *S. aureus.* In addition, coptisine can reduce the adhesion of *S. aureus* to human lung epithelial cells based on the result of plate colony counting assay. Molecular dynamics simulation revealed that coptisine can bind to the active pocket of SrtB, leading to its activity loss. Through the calculation of binding free energy between ligand and protein, site-directed mutagenesis and fluorescence spectroscopy quenching methods, it was confirmed that residues of Arg115, Asn116, and Ile182 played a vital role in the interaction of SrtB with coptisine. These data provide the theoretical basis for the therapy option to the infections caused by *S. aureus.*

## 1. Introduction

The rise of antibiotic-resistant bacteria has become an increasingly serious global public health issue [1,2]. An anti-virulence strategy, which is to restrain the virulence factors of bacterial rather than killing them directly to achieve the therapeutic effect, is expecting to be a new effective therapeutics strategy beyond antibiotics [3,4].

The sortase family plays vital role in *S. aureus* infection, and both sortaseA (SrtA) and sortaseB (SrtB) are important members of the family [5]. Research about the role and inhibitors of SrtA is adequate. Nevertheless, the role of SrtB in infection has been explored and is continuously being discovered, and the inhibitors reports relative to SrtB are lacking.

Based on the reports, SrtB plays a crucial role in the pathogenicity of *S. aureus*, and it anchors proteins containing the C-terminal sorting signal of the NPQTN motif to the bacterial cell wall [6]. Firstly, it cleaves the amino acid residue between T and N to form an acyl intermediate; subsequently, the amino group of pentaglycine cross-bridges that has been anchored to the lipid II peptidoglycan dissolves the intermediate and generates an amide bond of IsdC and lipid II. Finally, the amide bond is incorporated the into cell wall [7]. IsdC, a critical speed-limited protein during the capture of iron from host cells to guarantee the bacteria get enough free-iron to achieve a successful infection, is an identified substrate of SrtB [8,9,10].

Many research works have been done to explore the role of SrtB in the pathogenesis of *S. aureus*, and the results have shown that SrtB had a critical effect on the infection of *S. aureus* [6,7,11,12]. Mazmanian et al. [9] reported that, in a mouse infection model assay, mice were inoculated with wild-type *S. aureus* and its isogenic knockout mutant of SrtB, respectively, and they revealed that, compared with the wild-type group, the SrtB mutant strain group showed better survival rates, much smaller weight losses, significantly lower inflammatory response, fewer colony count in joints and kidneys, significant difference in clinical severity of arthritis, and a defect in the establishment of murine arthritis, indicating that SrtB played a vital role in the murine arthritis. The 3D structure of SrtB with its inhibitor was later revealed by Zong et al. along with the active site constituent and the catalytic machinery of SrtB with the inhibitor [7], which also providing the basis for the further study of SrtB with its novel inhibitors. After that, Ki-Bong Oh et al. performed a fibronectin-binding assay with *S. aureus* and its SrtB mutant. The result revealed that the adhesion of SrtB mutant strain to fibronectin was significantly reduced compared to the wild-type group [11], giving us the information that SrtB may also play a role in the adhesion of *S. aureus* to host cells during the infection. Subsequently, the 3D structure of SrtB with its NPQTN sorting signal substrate was reported by Jacobitz et al., who analyzed the mechanism of interaction between SrtB and its substrate, determined the active center and the major active-site amino acid residues with the methods of computational modeling, molecular dynamics simulations, and targeted amino acid mutagenesis [12], providing useful information for us to study the mechanism of SrtB with its inhibitors. All these reports indicated that SrtB might also be an important virulence factor of *S. aureus* participating in the establishment and persistence of *S. aureus* infections. Although these results are exciting in order for us to know the role of SrtB, there are still unknown parts of it [6]; therefore, working to explore the unknown part of SrtB is vital in future study.

Considering the crucial role of SrtB in the pathogenesis of *S. aureus*, it may be a potential drug target to combat the infections caused by this pathogenic bacteria. Unfortunately, there are few related reports of SrtB inhibitors. Therefore, discovering novel inhibitors of SrtB and clarifying the interaction mechanism between them can provide new therapeutic agents to *S. aureus* infecton.

Coptisine is a natural isoquinoline alkaloid compound that has many biological functions and has been demonstrated to have a therapeutic effect on many clinical diseases such as cancer [13,14], Alzheimer’s [15], cardiovascular and neurodegenerative diseases [16,17], hypercholesterolemic [18], and inflammatory response [19,20], all of which contributed to coptisine receiving lots of attention.

Here, we reported that coptisine can inhibit the activity of SrtB significantly in vitro, but does not exhibit antibacterial activity under the given conditions. Cytotoxicity and adhesion assays were performed to indicate that coptisine can protect human lung epithelial cells from *S. aureus* and can reduce the adhesion of *S. aureus* to human lung epithelial cells. Furthermore, we determined the interaction mechanism between coptisine and SrtB by molecular dynamics simulation, site-directed mutagenesis and fluorescence spectroscopy quenching methods.

## 2. Results and Discussion

### 2.1. Coptisine Inhibits the Activity of SrtB_Δ30_

**Figure 1:**
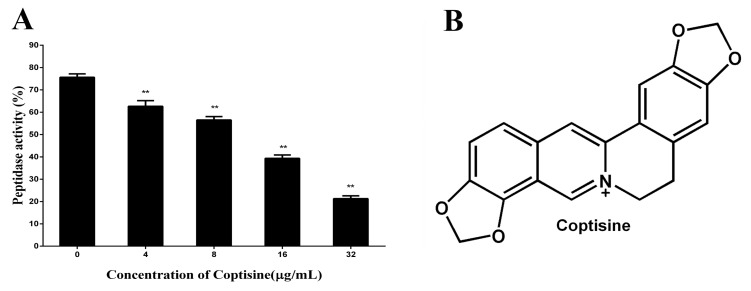
(**A**) the inhibitory effect of coptisine against SrtB_Δ30_. The data were present as mean ± SD, ** indicates *p* < 0.01 compared with the control group; (**B**) the chemical structure of coptisine.

In this paper, the inhibitory effect of coptisine to SrtB_Δ30_ was firstly determined by the change of fluorescence intensity. As shown in Figure 1A, compared with the control group, the activity of SrtB_Δ30_ attenuates observably with the increasing concentrations of coptisine (the IC50 was 8.74 µg/mL), indicating that coptisine can inhibit the activity of SrtB_Δ30_ with dose-dependency. The chemical structure of coptisine was shown in Figure 1B.

### 2.2. Coptisine Did Not Exhibit Antibacterial Activity against *S. aureus*

**Figure 2:**
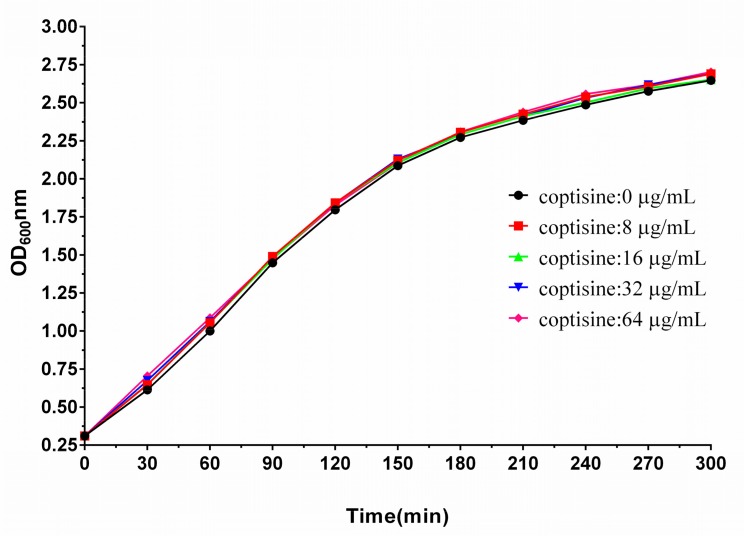
The growth kinetics of *S. aureus* 29213 with various concentrations of coptisine in the cultural medium. The data were present as mean ± SD (*n* = 3).

As shown in Table 1, four strains of *S. aureus* were used to assess the antimicrobial activities of coptisine against *S. aureus*. Interestingly, we found that coptisine did not exhibit antibacterial activity to *S. aureus* when the concentration of coptisine reached 1024 µg/mL. Seen from Figure 2, the growth trends of *S. aureus* almost corresponded with different concentrations of coptisine (0, 8, 16, 32, 64 µg/mL), suggesting that coptisine did not inhibit the growth of *S. aureus* under the given conditions of this study.

### 2.3. Coptisine Protects Human Lung Epithelial Cells from *S. aureus*

Consequently, based on the above-mentioned results, it was inferred that coptisine can protect human lung epithelial cells from *S. aureus*. The release of lactate dehydrogenase (LDH) was an important index of the cell membrane integrity and was widely used to analyze the toxicity of inhibitor to the cell. By measuring the LDH activity of the medium, we can achieve the quantitative analysis of cell toxicity.

**Figure 3:**
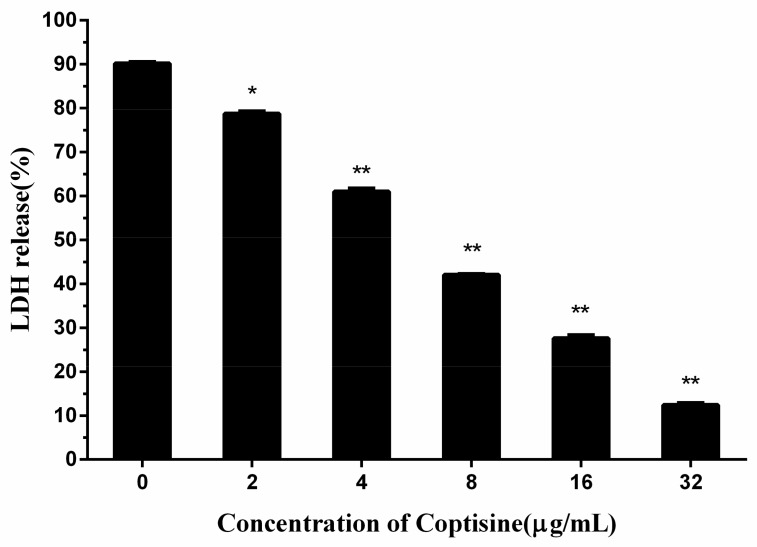
The lactate dehydrogenase (LDH)release of A549 cells by co-incubating with *S. aureus* and various concentrations of coptisine. The data were present as mean ± SD (*n* = 3). * indicates *p* < 0.05 and ** indicates *p* < 0.01.

As seen from Figure 3, the LDH release in the medium was reduced significantly with the rise of the coptisine content, when the concentration of coptisine reached 32 µg/mL, the LDH release reduced to 12.41%, showing a significant difference compared with the 90.19% of 0 µg/mL, indicating that coptisine can protect human lung epithelial cells from *S. aureus.*

### 2.4. Coptisine Reduced the Adhesion of S. aureus to Human Lung Epithelial Cells

**Figure 4:**
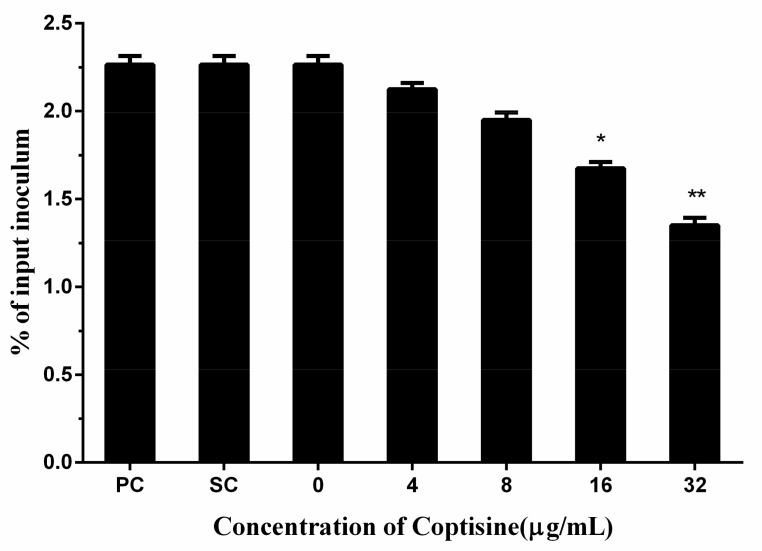
The adhesion of *S. aureus* to human lung epithelial cells after being treated with various concentrations of coptisine. The data were present as mean ± SD (*n* = 3). * indicates *p* < 0.05 and ** indicates *p* < 0.01. The results were presented as the percentage of colonies adhered to A549 cells and the initial inoculums. PC means potisve control, SC means solvent control.

Adhesion to host cells is a fundamental step of the pathogenic bacterium to achieve a successful infection. Therefore, adhesion has a crucial effect on the infections of pathogenic bacteria. In this study, we found that coptisine can reduce the adhesion of *S. aureus* to human lung epithelial cells with dose-dependency, and a significant difference was detected when the concentration of coptisine reached 16 µg/mL compared with the 0 µg/mL (Figure 4).

### 2.5. Determine the Binding Mode of Coptisine with SrtB_Δ30_

**Figure 5:**
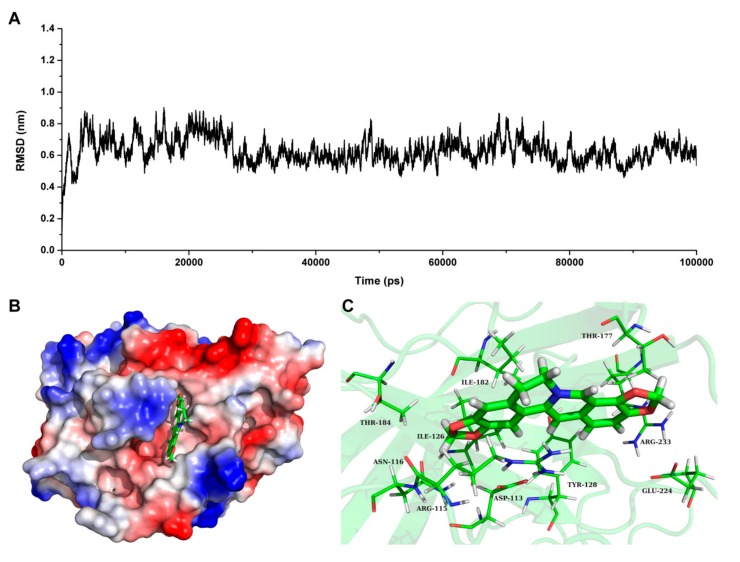
The information of interaction between coptisine and SrtB_Δ30_ based on the MD simulation. (**A**) the root mean square deviation (RMSD) revealed by the backbone atoms of the protein during molecular dynamics simulations of SrtB_Δ30_-coptisine; (**B**) the stable binding mode of coptisine with SrtB_Δ30_ based on the MD simulation; and (**C**) the detail binding sites in the SrtB_Δ30_-coptisine complex.

Based on the result of SrtB_Δ30_ activity assay, we speculate that coptisine can interact with SrtB_Δ30_ directly. To investigate the information about the interaction between ligand and protein, molecular docking and molecular modelling were performed for the SrtB_Δ30_-coptisine complex system. During the 100-ns simulation, the 3D structure of SrtB_Δ30_-coptisine complex was determined. Seen from Figure 5A, the root mean square deviations of the protein confirmed that the SrtB_Δ30_-coptisine system reached equilibrium at 30 ns, indicating that the structure of SrtB_Δ30_-coptisine was reliable. Figure 5B has clearly shown coptisine bound to the active pocket of SrtB_Δ30_ via non-covalent interactions, which is the catalytic reaction region of the substrate NPQTN motif. In detail, Arg115 and Asn116 were close to the conjugate ring group of the left side in coptisine, leading to the formation of strong interactions between the two residues and coptisine (Figure 5C). In addition, residues Asp113, Tyr128, and Ile182 were closer to the central section of coptisine than to the other residues, suggesting that the central section of coptisine was fixed by Asp113, Tyr128 and Ile182. Finally, the conjugate ring groups on the right side of coptisine showed a relatively closer distance to Thr177, Glu224 and Arg233. Therefore, a strong interaction can form between coptisine and Asp113, Tyr128 and Ile182.

**Figure 6:**
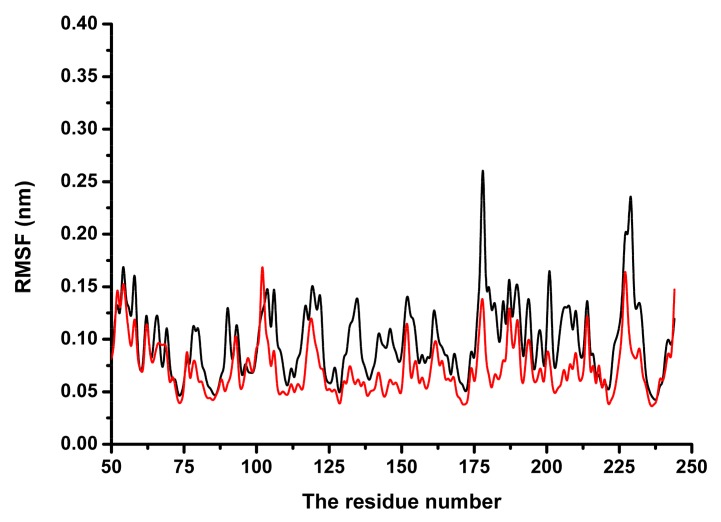
The RMSF of residues of SrtB_Δ30_ in the complex (red line) and free protein (black line) was shown.

Subsequently, the conformational changes in the residue side-chains before and after binding inhibitor were investigated by analyzing the root mean square fluctuations. Figure 6 indicates that the flexibility of the residues bound to coptisine differed from those of residues in the free protein. The flexibility of the residues in the binding sites of SrtB_Δ30_ that bound with the inhibitor was smaller than those in the free protein, with the root mean square fluctuations < 0.4 nm. These results indicated that the flexibilities of these amino acid residues were restrained (more rigid) after binding to coptisine.

These results demonstrated that residues Asp113, Arg115, Asn116, Tyr128, Thr177, Ile182, Glu224 and Arg233 play a critical role in maintaining the stability of the binding of coptisine with SrtB_Δ30_.

### 2.6. Determination of the Binding Sites of Coptisine with SrtB_Δ30_

To acquire the detailed information about the contribution of residues to the binding of coptisine with SrtB_Δ30_, the Molecular Mechanics Poisson–Boltzmann Surface Area (MM-PBSA) method was performed to calculate the binding free energy between ligand and protein in the complex system.

**Figure 7:**
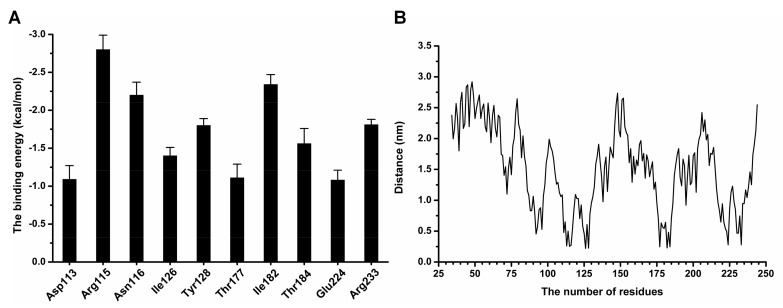
(**A**) the binding energies decomposition of residues at the binding sties among SrtB_Δ30_ with coptisine; (**B**) the distance analysis between all of the residues of SrtB_Δ30_ and coptisine.

**Figure 8:**
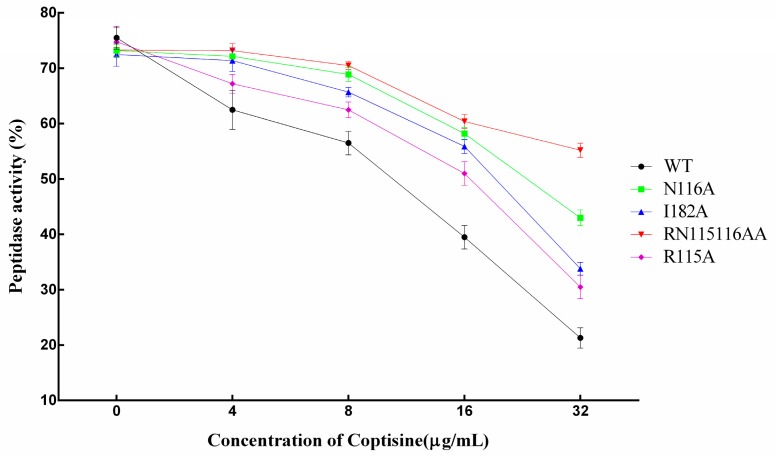
The activity inhibit of coptisine to the mutant proteins in vitro. The data were present as mean ± SD (*n* = 3).

Figure 7A clearly shows that Arg115 and Asn116 had the stronger binding energy with coptisine, with ∆*E_total_* values of −2.8 and −2.2 kcal/moL, respectively, indicating that Arg115 and Asn116 formed strong interactions with the conjugate ring group of the left side in coptisine. Moreover, Tyr128 and Ile182 also showed large contributions to the binding free energy, with ∆*E_total_* values of −1.8 and −2.34 kcal/moL, indicating that the two residues can stabilize coptisine through the strong interaction with the central section of coptisine. Additionally, the ∆*E_total_* values of Thr177, Glu224 and Arg233 less than −1.00 kcal/moL indicate an interaction between Thr177, Glu224, Arg233 and the right side of coptisine. As shown in Figure 7B, the distances between all residues and coptisine were calculated during the last 60 ns of the simulation. Figure 7B also shows that the distances of residues with higher binding energies to coptisine were lower, with the values less than 0.25 nm, which agrees with the above results. Therefore, according to the results revealed in Figure 8, the residues Arg115, Asn116, Tyr128, Thr177, Ile182, Glu224 and Arg233 are likely critical for the binding of coptisine with SrtB_Δ30_.

To verify the above results, molecular dynamics simulation was carried out for the complexes of R115A, N116A and I182A mutants with coptisine. The binding free energies between mutants and ligand were also calculated by the MM-PBSA method. Meanwhile, the binding constants (*K_A_*) of the complexes mentioned above were analyzed with the method of fluorescence spectroscopy quenching [21,22]. The binding energies of mutants with coptisine shown in Table 2 were weaker than that of SrtB_Δ30_-WT with coptisine (−11.5 kcal/moL for R115A, −13.2 kcal/moL for N116A, −10.9 kcal/moL for I182A). On the other hand, based on the data of the experiment, the binding constants (*K_A_*) of the ligand with the mutant proteins were lower, with the order as follows: WT > N116A > R115A > I182A, showing a high consistency to the theoretical calculation results. Therefore, it is convincing that the binding model of SrtB_Δ30_ with coptisine was reliably obtained from MD simulation.

In summary, coptisine can bind to the catalytic pocket of SrtB_Δ30_, and residues of Arg115, Asn116, Tyr128, Thr177, Ile182, Glu224 and Arg233 play a critical role during the binding process. Due to the binding of coptisine with the catalytic pocket of SrtB_Δ30_, the biological activity of SrtB was inhibited.

In addition, we found that the mutants also have bioactivity compared with WT-SrtB. However, the inhibitory effect of coptisine on mutants was obviously weaker than to WT-SrtB. Figure 8 clearly shows that the inhibitory effect of coptisine on mutants was lower, with the order as follows: WT > R115A > I182A > N116A > RN115116AA, suggesting that all three of the residues play a key role in the interaction between coptisine and SrtB, and the results on the basis of molecular modelling were convincing.

### 2.7. Discussion

The rise of antibiotic-resistant and other multi-drug resistant strains of *S. aureus* have caused a tremendous threat to global public health [23,24]. New effective therapeutics strategies beyond antibiotics are urgently needed [25,26]. One promising therapeutic scheme taking a virulence factor as the target is being increasingly explored. Different from the traditional strategies that are aimed at killing bacteria, this new therapeutic scheme can diminish the rate of development of bacterial resistance. Importantly, anti-virulence drugs would apply a milder evolutionary pressure on the target bacterium, and would not cause the development of drug-resistance bacteria like antibiotics [27]. In addition, adhesion to the host tissue is a fundamental step for pathogenic bacteria colonization and infection and further pathogens including *S. aureus*. Antiahesion strategy can also be a potential alternative therapeutic agent to overcome the global threat of the antibiotic resistance of *S. aureus* [28].

It has been demonstrated that SrtB as a virulence factor of *S. aureus* plays a vital role in the invasion and infection, while there are few reports about SrtB inhibitors. In this study, we found that coptisine, a natural compound, can inhibit the activity of SrtB in vitro but did not exhibit antibacterial activity under the given conditions.

Methicillin-resistant *S. aureus* (MRSA) has become one of the leading etiologies of nosocomial pneumonia as a result of an increase in *S. aureus* infections caused by methicillin-resistant strains [29]. The prevalence of community-acquired MRSA pneumonia, which historically affects younger patients is also increasing [29]. Even with early diagnosis and appropriate treatment, MRSA pneumonia still carries an unacceptably high mortality, mortality rate and costs [29,30,31].

In order to investigate the effect of coptisine on *S. aureus* pneumonia, we chose A549 cells, a human lung epithelial cell mode, as the cell mode to perform the cytotoxicity and adhesion assays. It was confirmed that coptisine can protect human lung epithelial cells from *S. aureus* and can reduce the adhesion of *S. aureus* to human lung epithelial cells.

Furthermore, molecular dynamics simulation, site-specific mutagenesis and fluorescence spectroscopy quenching methods were performed to determine the binding model and inhibitory mechanism of coptisine to SrtB. On the basis of the 100-ns simulation, we believed that coptisine can bind to the catalytic pocket of SrtB, and Arg115, Asn116, Tyr128, Thr177, Ile182, Glu224 and Arg233 residues play a critical role in the binding of coptisine with SrtB. This result was confirmed by site-specific mutagenesis and the fluorescence spectroscopy quenching method. The experimental data are in good agreement with the results gained from the theoretical calculation. Due to the binding of coptisine with the catalytic pocket in SrtB, the binding of SrtB with the substrate, the sorting signal NPQTN motif, was blocked, causing the weakening of the catalytic activity of SrtB, which was supported by fluorescence resonance energy transfer (FRET) assay. The experimental data show that mutants also have bioactivity compared with WT-SrtB (Figure 8). However, the inhibitory effect of coptisine on mutants was weaker obviously than on WT-SrtB. These results indicate that the catalytic activity of SrtB can be effectively depressed by the binding of coptisine with the catalytic pocket in SrtB. According to the results above, it is convincing that coptisine can be a new potential candidate of therapeutics to combat *S. aureus*.

## 3. Materials and Methods

### 3.1. Materials

Coptisine (purity > 98%) was purchased from Shanghai Yuanye Co., Ltd. (Shanghai, China) and was dissolved in dimethyl sulfoxide (DMSO, Sigma-Aldrich, St. Louis, MO, USA). Fluorescent substrate peptide Dabcyl-QANPQTNEE-Edans was purchased from Shanghai GL Biochem Co., Ltd. (GL Biochem, Shanghai, China).

### 3.2. Cloning, Expression and Purification of SrtB_Δ30_ and Its Mutants

*S. aureus* ATCC 29213 genomic DNA was used as the template to amplify the DNA sequence of SrtB_Δ30_; the fragment was cloned into pET-28a vector after being digested with BamHI and XhoI and transformed into *E. coli* BL21 (DE3).

The mutant sequences were amplified by using the Quick Change site-directed mutation kit (Stratagene, La Jolla, CA, USA) with pET-28a plasmid as the template. Then, the plasmids carrying the gene of the mutants were transformed into *E. coli* BL21 (DE3) after being digested with the DpnI enzyme. The primers pairs used in this assay were shown in Table 3.

*E. coli* BL21 (DE3) was cultured in Luria–Bertani (LB) media containing 50µg/L Kana. IPTG was added into the medium to reach a final concentration of 0.5 mM when the OD_600_ reached 0.8 and continued to incubate for 16 h at 16 °C with shaking. The cell was harvested by being centrifuged for 30 min with 4000 rpm at 4 °C and then suspended into the lysis buffer and lysed ultrasonically. The lysate supernatant was loaded into a His-affinity column (GE Healthcare Life Sciences, QIAGEN GmbH, Germany) after being centrifuged for 30 min with 12,000 rpm at 4 °C. The contaminant proteins were eliminated with 20 mM imidazole in the equilibration buffer (NaCl 300 mM, NaH_2_PO_4_ 50 mM, Tris 10 mM, pH 8.0), SrtB_Δ30_ and the mutant proteins were eluted with 300 mM imidazole in the equilibration buffer. The proteins were concentrated at 4 °C and stored at −20 °C after analyzed by SDS-PAGE.

### 3.3. Activity Inhibit Assay

The inhibitory effect of coptisine to SrtB_Δ30_ was performed according to the fluorescence resonance energy transfer (FRET) method reported previously [32,33] with some amendments. The assay mainly involves two procedures and was carried out in a black 96-well plate. The volume of the reaction system was 100 μL in total, mainly containing the buffer (Tris 50 mM, NaCl 150 mM, pH 7.5), coptisine (various concentrations), SrtB_Δ30_ protein and fluorescent peptide substrate (GL Biochem, Shanghai, China). The molar ratio of the protein and the substrate was 1:3. Firstly, SrtB_Δ30_ with specified concentrations coptisine in the buffer were incubated for 30 min at 37 °C. Subsequently, fluorescent peptide substrate was added into the reaction system and incubated continuously for 60 min at 37 °C. The fluorescence intensity was measured with emission wavelengths of 350 nm and excitation wavelengths of 520 nm. Proteinase K with fluorescent peptide substrate and buffer were taken as the positive control, and the SrtB buffer was taken as the negative control. The same method was used to assess the inhibition effect of coptisine on the mutant proteins.

### 3.4. Minimal Inhibitory Concentration (MIC) and Growth Curves

#### 3.4.1. MIC

The minimal inhibitory concentration (MIC) of coptisine against *S. aureus* were assessed based on the broth microdilution method of the National Committee for Clinical Laboratory Standards (NCCLS). In brief, bacteria cultured overnight was diluted (1:100) to fresh TSB medium and further cultured for 4 h at 37 °C with shaking. In a 96-well plate, various concentrations of coptisine (1024 µg/mL to 0.5 µg/mL) were prepared, and the top concentration was 1024 µg/mL. Two-fold serial dilutions were performed to get the gradient concentration. Then, bacteria was added into each well to get a final density of 5 × 10^5^ cfu/mL. The plate was cultured at 37 °C for 24 h to determine the minimal inhibitory concentration. Oxacillin was used as a positive control.

#### 3.4.2. Growth Curves

Bacteria cultured overnight was diluted (1:100) to fresh TSB medium and further cultured to OD_600_ reached to 0.3, followed by being sub-packaged to fresh TSB medium with expectancy concentrations of coptisine (0, 8, 16, 32, 64 μg/mL), recording OD_600_ every 30 min until reaching stationary phases.

### 3.5. Cytotoxicity Assay Mediated by S. aureus

Based on the method described previously [34] with some modifications, human lung epithelial A549 cells (ATCC) were cultured with complete DMEM medium (DMEM with 10% fetal bovine serum and 100 IU/mL of penicillin and streptomycin) at 37 °C with 5% CO_2_. After being dispersed with 0.25% trypsin (0.02% EDTA was contained), the cells were suspended into antibiotic-free cultural media (DMEM with 10% fetal bovine serum). Then, the cells were seeded into a 96-well plate (Corning Costar, Corning, NY, USA) at 2 × 10^4^ per well and cultured for another 18 h at 37 °C with 5% CO_2_.

*S. aureus* grown to a mid-logarithmic phase were harvested and suspended into DMEM with given concentrations (0, 2, 4, 8, 16, 32 µg/mL) of coptisine, and bacterial density was adjusted to 4.2 × 10^7^ cfu/mL through controlling the value OD_600_. The medium of A549 cells was replaced with the suspension of bacterial and coptisine and kept co-incubated for 5 h at 37 °C with 5% CO_2_. Cell viability was determined by measuring the lactate dehydrogenase (LDH) release at 490 nm with a Cytotoxicity Assay Kit (LDH) (Roche, Basel, Switzerland). A549 cells treated with 0.2% Triton X-100 were taken as the positive control, and those treated with DMEM only were taken as the negative control. The same amount of DMSO that corresponds to each concentration of coptisine was taken as the solvent control. All of the procedures were carried out by following the specifications.

### 3.6. Adhesion Assay Mediated by S. aureus

According to a method reported previously [35] with some modifications, human lung epithelial A549 cells (ATCC) were cultured with complete DMEM medium (DMEM with 10% fetal bovine serum and 100IU/mL of penicillin and streptomycin). Then, the cells were dispersed with 0.25% trypsin (0.02% EDTA was contained) and suspended into antibiotic-free cultural media (DMEM with 10% fetal bovine serum). Subsequently, the cells were plated into a 24-well plate with 2.5 × 10^5^ per well and were kept incubated for 18 h at 37 °C with 5% CO_2_.

*S. aureus* grown to a mid-logarithmic phase were harvested and suspended into DMEM with given concentrations (0, 4, 8, 16, 32 µg/mL) of coptisine, and bacterial density was adjusted to 2 × 10^6^ cfu/mL by controlling the value OD_600_. The medium of A549 cells was replaced with the suspension of bacterial and coptisine. After incubating for 2 h at 37 °C with 5% CO_2_, the culture medium was removed, and the cells were washed with sterile PBS three times. Subsequently, 200 µL 0.25% trypsin (0.02% EDTA was contained) was added to disperse the A549 cells by treating them for 2 min. Then, 800 µL 0.2% Triton X-100 was added into the system, and the cells were harvested after eddying for 5 min. The amount of the bacteria adhered to A549 cells was determined by the plate colony counting method after gradient dilution. Cells treated with bacteria but without coptisine and DMSO were taken as the positive control, and those treated with DMEM only were taken as the negative control. Cells treated with bacteria and an equal amount of DMSO corresponding to 32 µg/mL of coptisine were taken as the solvent control.

### 3.7. Molecular Simulation

The crystal structure of SrtB (PDB code: 1NG5) was used to perform the molecular docking with the Auto Dock 4.0 software (Molecular Graphics Laboratory, The Scripps Research Institute, La Jolla, CA, USA). The 3D structure of coptisine was optimized by a Gaussian 09 program (Gaussian Inc., Wallingford, CT, USA) at the B3LYP/6-311G* level. The complex structure obtained from molecular docking was used as the original structure to carry out the molecular dynamics simulation. The detailed processes and methods are described in the Supplementary Materials.

### 3.8. Statistical Analysis

The experimental data were shown as mean ± SD, the significance of difference was analyzed according to the two-tailed Student’s *t*-tests, and the statistically significant was determined when *p* < 0.05.

## 4. Conclusions

Coptisine, a natural compound, can alleviate the damage of human lung epithelial A549 cells caused by *S. aureus* significantly, and can reduce the adhesion of *S. aureus* to A549 cells by targeting at an important virulence factor SrtB of *S. aureus*. The experimental data show that coptisine can inhibit the activity of SrtB dramatically in vitro, more interestingly, it did not exhibit antibacterial activity under the given condition, further demonstrated that the protective effect to A549 cells were based on the inhibitory effect to SrtB. Furthermore, the results of molecular dynamics simulation indicate that coptisine can bind to the catalytic pocket of SrtB, blocking the binding of SrtB with its substrate, causing the activity loss of SrtB. After site-specific mutagenesis, the inhibitory effect of coptisine on mutants was weaker than on WT-SrtB, confirming that the results of molecular dynamics simulation were convinced. In conclusion, we get a natural compound coptisine via targeting at SrtB of *S. aureus* to achieve protect effect to human lung epithelial A549 cells, and determined the inhibitory mechanism of coptisine to SrtB, providing theoretical basis and new therapeutic strategy to combat the infections caused by *S. aureus*.

## Figures and Tables

**Table 1:** The antimicrobial activities of coptisine against *S. aureus*.

Inhibitors	Strains			
	29213	BAA-1717	BAA-1707	8325-4
Coptisine (µg/mL)	>1024	>1024	>1024	>1024
Oxacillin (µg/mL)	0.25	128	64	0.125

**Table 2:** The calculated binding free energy (kcal/moL) and the binding constants (*K_A_*) gained from the fluorescence spectroscopy quenching assay of proteins (WT-SrtB_Δ30_, R115A, N116A, and I182A) with coptisine.

	WT-SrtB	R115A	N116A	I182A
Computational Method	−19.4 ± 2.6	−11.5 ± 2.1	−13.2 ± 2.3	−10.9 ± 2.4
*K_A_* (1 × 10^4^) L·mol^−1^	13.6 ± 2.4	8.3 ± 1.9	9.2 ± 2.2	7.8 ± 1.8

**Table 3:** Primers used in this study.

Name	Oligonucleotide (5′-3′) ^a^
SrtB_Δ30_-F	CGCGGATCCGAAGACAAGCAAGAACGCGC
SrtB_Δ30_-R	CCGCTCGAGTTAACTTACCTTAATTATTTTTGCGACAAC
SrtB_Δ30_-R115F	GGTAGTATTTTTATGGATTTTGCGAATGAATTGAAGAATTTAAATC
SrtB_Δ30_-R115R	GATTTAAATTCTTCAATTCATTCGCAAAATCCATAAAAATACTACC
SrtB_Δ30_-N116F	GTATTTTTATGGATTTTAGAGCGGAATTGAAGAATTTAAATC
SrtB_Δ30_-N116R	GATTTAAATTCTTCAATTCCGCTCTAAAATCCATAAAAATAC
SrtB_Δ30_-I182F	CTACTACTAAAGATAATTACGCGCGTACAGATTTTGAAAATG
SrtB_Δ30_-I182R	CATTTTCAAAATCTGTACGCGCGTAATTATCTTTAGTAGTAG
SrtB_Δ30_-RN115116F	GTAGTATTTTTATGGATTTTGCGGCGGAATTGAAGAATTTAAATC
SrtB_Δ30_-RN115116R	GATTTAAATTCTTCAATTCCGCCGCAAAATCCATAAAAATACTAC

^a^ The underlined basic group represents restriction endonuclease recognition sites or mutated codons.

## Supplementary Materials

The following are available online.
